# Single-Nucleus Transcriptome Profiling from the Hippocampus of a PTSD Mouse Model and CBD-Treated Cohorts

**DOI:** 10.3390/genes15040519

**Published:** 2024-04-21

**Authors:** Guanbo Xie, Yihan Qin, Ning Wu, Xiao Han, Jin Li

**Affiliations:** State Key Laboratory of Toxicology and Medical Countermeasures, Beijing Key Laboratory of Neuropsychopharmacology, Beijing Institute of Pharmacology and Toxicology, Beijing 100850, China; xiegb666@163.com (G.X.); qinyihan1999@163.com (Y.Q.); wuning7671@126.com (N.W.); jinli9802@163.com (J.L.)

**Keywords:** post-traumatic stress disorder, cannabidiol, single-nucleus RNA sequencing, hippocampus, excitatory/inhibitory neurons

## Abstract

Post-traumatic stress disorder (PTSD) is the most common psychiatric disorder after a catastrophic event; however, the efficacious treatment options remain insufficient. Increasing evidence suggests that cannabidiol (CBD) exhibits optimal therapeutic effects for treating PTSD. To elucidate the cell-type-specific transcriptomic pathology of PTSD and the mechanisms of CBD against this disease, we conducted single-nucleus RNA sequencing (snRNA-seq) in the hippocampus of PTSD-modeled mice and CBD-treated cohorts. We constructed a mouse model by adding electric foot shocks following exposure to single prolonged stress (SPS+S) and tested the freezing time, anxiety-like behavior, and cognitive behavior. CBD was administrated before every behavioral test. The PTSD-modeled mice displayed behaviors resembling those of PTSD in all behavioral tests, and CBD treatment alleviated all of these PTSD-like behaviors (n = 8/group). Three mice with representative behavioral phenotypes were selected from each group for snRNA-seq 15 days after the SPS+S. We primarily focused on the excitatory neurons (ExNs) and inhibitory neurons (InNs), which accounted for 68.4% of the total cell annotations. A total of 88 differentially upregulated genes and 305 differentially downregulated genes were found in the PTSD mice, which were found to exhibit significant alterations in pathways and biological processes associated with fear response, synaptic communication, protein synthesis, oxidative phosphorylation, and oxidative stress response. A total of 63 overlapping genes in InNs were identified as key genes for CBD in the treatment of PTSD. Subsequent Gene Ontology (GO) and Kyoto Encyclopedia of Genes and Genomes (KEGG) analyses revealed that the anti-PTSD effect of CBD was related to the regulation of protein synthesis, oxidative phosphorylation, oxidative stress response, and fear response. Furthermore, gene set enrichment analysis (GSEA) revealed that CBD also enhanced retrograde endocannabinoid signaling in ExNs, which was found to be suppressed in the PTSD group. Our research may provide a potential explanation for the pathogenesis of PTSD and facilitate the discovery of novel therapeutic targets for drug development. Moreover, it may shed light on the therapeutic mechanisms of CBD.

## 1. Introduction

Post-traumatic stress disorder (PTSD) is the most prevalent psychiatric disorder that occurs following major catastrophic events [1,2]. Nowadays, with the emergence of catastrophes such as the COVID-19 pandemic, wars, and other related events, the global incidence of PTSD has witnessed a significant increase on a large scale [1,3,4]. The symptom cluster of PTSD exhibits heterogeneity, and individuals with PTSD often experience various comorbidities, such as anxiety, depression, cognitive impairments, peripheral inflammation, cardiovascular problems, and metabolic disorders [5,6,7,8]. Despite the significant burden that PTSD imposes on individuals and society, there are currently no recognized or established molecular biomarkers for the diagnosis of this disease.

A growing corpus of studies established a connection between an imbalance in the regulation of fear memory and PTSD. Imbalances within the neural circuitry responsible for fear response, particularly in regions such as the medial prefrontal cortex, amygdala, and hippocampus, contribute to flawed processing and understanding of distressing experiences, thereby facilitating the onset and persistence of PTSD. Within the ensemble of brain areas, the hippocampus stands out as a vital component, as it is known to significantly influence both memory functioning and emotional control [9,10]. In a traumatic event, the hippocampus is implicated in contextual fear learning, fear memory consolidation, and retrieval [11,12,13]. Studies on PTSD patients revealed that they may experience a reduced hippocampal volume, which was considered a common feature of structural changes in the brains of individuals with PTSD [14,15,16]. Gaining insights into the molecular foundations of fear learning and memory within the hippocampus after traumatic experiences is essential for unraveling the etiology of PTSD and developing interventions for treating this disease.

Current pharmacotherapies for PTSD predominantly involve the utilization of selective serotonin reuptake inhibitors, which exhibit suboptimal treatment efficacy. Studies from our lab and others have shown that cannabidiol (CBD) alleviates the main PTSD-like behaviors in rodents. However, the molecular mechanism of action for the treatment of PTSD with CBD is still being investigated due to its multiple target sites, which hinder its clinical translation. An in-depth investigation of the molecular mechanism underlying CBD’s anti-PTSD effects is desperately needed.

In recent years, several studies on the transcriptomics of PTSD have been conducted, revealing a potential association among neuronal signaling, neurogenesis, inflammation, synaptic plasticity, and the development of this disorder [17,18,19]. However, these studies lack cell-type-specific interpretations. The varied presentation of PTSD symptoms implies that its etiology may be diverse; hence, animal models that accurately replicate the diverse range of symptoms associated with PTSD are considered optimal for investigating its pathogenesis and assessing the efficacy of pharmaceutical interventions. Within our investigation, we employed a modified single prolonged stress plus shock (SPS+S) mouse model, which was adapted from an SPS PTSD model in rats that posed challenges for replication in mice. We added a minor shock after the SPS procedures and tested the freezing behavior, anxiety-like behavior, and cognitive behavior in the model, which encompassed the majority of human PTSD symptom clusters. The advancement of single-cell or single-nucleus RNA sequencing (snRNA-seq) methodologies facilitates an in-depth examination of the transcriptome at an unprecedented cellular level, allowing distinct disease-associated expression patterns within individual cell types to be discovered. Here, we explored cell-type-specific mechanisms in the hippocampus of PTSD mice and investigated the effects, potential therapeutic targets, and molecular mechanisms of CBD treatment by performing single-nucleus gene expression profiling in the SPS+S mouse model.

## 2. Materials and Methods

### 2.1. Animals

Eight-week-old male C57BL/6J mice were obtained from SPF (Beijing) Biotech Co., Ltd. (Beijing, China). The exclusive use of males was intentional to prevent any possible interference of the female rodents’ estrous cycle with the hypothalamic pituitary adrenal axis. The animals were maintained under controlled conditions with a 12 h light–dark regimen (light period from 7:00 am to 7:00 pm), a constant temperature of 22 ± 2 °C, and a relative humidity between 40 and 60%. Prior to the commencement of the trials, a one-week acclimatization period was provided. Subsequently, the mice were randomly allocated into three groups: the control group, the model group, and the CBD group. Eight mice in each group were used for behavioral experiments, and three mice in each group were used for SnRNA-seq. The study was conducted according to the guidelines of the Declaration of Helsinki and was approved by the Institutional Review Board of the Beijing Institute of Pharmacology and Toxicology.

### 2.2. Behavioral Experiments

#### 2.2.1. SPS+S Model

**Figure 1:**
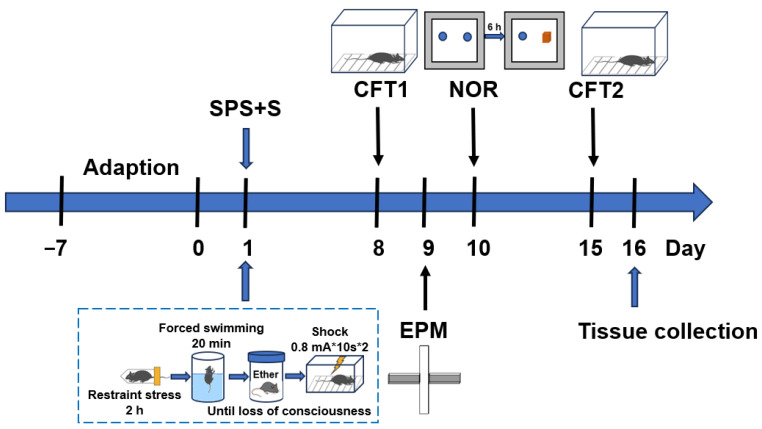
Experimental schedule. The SPS+S procedure was completed on day 1. After incubation (from days 1 to 7), various behavioral tests were conducted, including the contextual freezing test (CFT) on day 8 and day 15, the elevated plus maze (EPM) on day 9, the novel object recognition (NOR) test on day 9 and day 10, and tissue collection on day 16.

The SPS+S procedure was modified to consist of four stages [20]. First, each mouse was physically restrained for 2 h in a 50 mL Plexiglas tube with holes spaced 0.5 cm apart to allow it to breathe. The mouse was then immediately placed in a glass beaker (50 cm high, 24 cm diameter) filled with water (23 °C) to 2/3 of its height for 20 min of forced swimming. After a recovery duration of 15 min, every mouse was subjected to diethyl ether until it reached a state of unconsciousness. Following a 30 min recuperation period, the mice were subjected to two successive unavoidable electrical shocks, which had an intensity of 0.8 mA and a spacing interval of 10 s, and each shock lasted for 10 s; then, the mice remained for another 30 s in the shock chambers (AniLab Scientific Instruments, Ningbo, China). Mice that underwent these SPS+S procedures were referred to as the model group. For the control group, the mice were kept in their home cages throughout the SPS procedure and then placed in the shock chamber for the same amount of time as the model group but without shocks. All mice were allowed to remain undisturbed for 7 days prior to the behavioral tests. Figure 1 illustrates the experimental procedure for the SPS+S stressors and the behavioral test.

#### 2.2.2. Contextual Freezing Test (CFT)

The CFT was performed on day 8 and day 15 after SPS+S. In brief, the mice underwent a 5 min re-entry into the shock compartments, devoid of any shock administration. The aggregate freezing duration was quantified, and the percentage of freezing time during this 5 min assessment was documented using computerized software (AniLab Scientific Instruments, Ningbo, China) [21,22].

#### 2.2.3. Elevated plus Maze (EPM) Test

The EPM test is a widely used method for evaluating rodent behavior that is indicative of PTSD-related anxiety [23]. The cross-shaped apparatus is composed of four branching arms, which include two open arms (30 cm × 5 cm), two enclosed arms (30 cm × 5 cm × 15 cm), and a central area (30 cm × 5 cm), all positioned 50 cm above the ground. The experiment begins with the mouse being placed in the center, oriented toward the open arm, and permitted to roam without restriction for a duration of 5 min. Behavioral parameters, such as the duration spent in the open arm and the frequency of entry into all arms, were quantified using the VisuTrack software (version 1.0, Shanghai Xinruan Information Technology Co., Ltd., Shanghai, China). When all of the animal’s limbs entered either the open or closed arm, it was counted as one entry. The computed ratios—the time spent in the open arm relative to the total exploration period and the number of entries into the open arm compared to the cumulative arm entries—served as indices of the animal’s anxiety level.

#### 2.2.4. Novel Object Recognition (NOR) Test

The NOR test consisted of a training trial and a test trial. The initiation of each trial involved introducing the mouse into an NOR box (50 cm × 50 cm × 50 cm) and allowing it to freely explore for 5 min to habituate to the surroundings. During the training phase, the mouse was given 5 min to explore the box containing two matching objects placed equidistantly from the box walls. Following a 6 h interval after the training, the test trial ensued, where the same setup was used, except one of the familiar objects was substituted with an unfamiliar one. Throughout the 5 min test period, durations spent investigating both the familiar item (T1) and the novel item (T2) were recorded. The recognition index (RI), a measure of memory discrimination, was calculated as T1 divided by the sum of T1 and T2 [24]. An object was considered explored when the mouse touched, sniffed, or directed its face toward it at a proximity of 2 cm.

### 2.3. Drug Administration

Cannabidiol (verified to be 99.9% pure via high-performance liquid chromatography and devoid of any detectable phytocannabinoids; sourced from Hempson, Kunming, China) was dissolved in saline containing 2% dimethyl sulfoxide (DMSO) and 2% Cremophor EL for intraperitoneal (i.p.) injection at a dose of 10 mg/kg and was administered 30 min before each behavioral experiment. The selected dosage was based on the findings of prior studies [22,25,26,27,28]. The mice that underwent SPS+S procedures and CBD administration were referred to as the CBD group.

### 2.4. SnRNA-seq and Analysis

#### 2.4.1. Tissue Dissociation for 10× Genomics

The brains of the control, model, and CBD groups were removed on day 16. In order to mitigate variances among individual tissues and meet the requirements of sequencing analysis for cell numbers, three representative mice from each group were selected, and their hippocampi were extracted and mixed to form a sample. Therefore, there was one final sample for each group to undergo subsequent sequencing analysis. Tissue samples of the three groups were dissected, delicately dissociated with a pipette, and incubated in 0.05% trypsin diluted in PBS for 10 min. To neutralize the trypsin, 5% PBS was employed, followed by filtration through a 70 μm filter. A hemocytometer was utilized for the enumeration of individual cells. For single-cell sequencing, viable cells were selectively sorted. The subsequent step was the execution of snRNA-seq utilizing the Chromium Single-Cell 3′ Library and the Gel Bead & Multiplex Kit (version 3.1) from 10× Genomics. Sequencing was carried out on the Illumina Novaseq6000 platform, which was configured with parameters such as paired-end 150, dual indexing, and a minimum depth of 23,000 read pairs per cell. Raw sequencing data were archived in the GEO database under the accession number GEO: GSE. The Cell Ranger software pipeline (version 7.0.1) from 10× Genomics was used to deconvolute cellular barcodes, align reads to the genomic and transcriptomic references via the STAR aligner, and downsample reads as needed to produce normalized composite data across samples, thereby creating a gene count matrix for each cell.

#### 2.4.2. Quality Control and Cell Type Identification

By employing the Seurat R package (v4.3.0.1) [29], the dataset’s unique molecular identifier (UMI) count matrix underwent processing. To exclude low-quality and multiply captured cells, those with gene counts below 200 or over 10,000 or UMI counts under 1000 were removed. Additionally, cells displaying an over 30% mitochondrial gene content, which was indicative of poor quality, were also discarded. This quality control resulted in 31,866 single cells for further analysis, with 9793, 10,119, and 11,954 cells originating from the control, model, and CBD groups, respectively. To normalize the gene expression per cell based on the total expression, the log NormalizeData function was applied, followed by multiplication by a scaling factor of 10,000. The variable genes were identified using Seurat’s Find Variable Genes feature. A graph-based clustering method was implemented through the Find Clusters function, and cells were grouped according to their gene expression patterns. Visualization of these cells was achieved utilizing two-dimensional Uniform Manifold Approximation and Projection (UMAP) via Seurat’s RunUMAP function. Finally, Seurat’s Find All Markers function was employed to pinpoint signature genes for each cluster.

#### 2.4.3. SnRNA-seq Data Analysis

For cell type identification, the type of cell was identified based mainly on the marker genes from previous studies and the CellMarker database [29]. Genes with *p* < 0.05 and log2 fold change >0.25 were considered as significantly upregulated differentially expressed genes (DEGs). Genes with *p* < 0.05 and log2 fold change <−0.25 were considered as significantly downregulated DEGs. The overlapping genes from the downregulated genes of the model group relative to the control group and the upregulated genes of the CBD group relative to the model group were analyzed through Gene Ontology (GO) enrichment analysis and Kyoto Encyclopedia of Genes and Genomes (KEGG) analysis with the enrich GO function and enrich KEGG function, respectively, in the R package clusterProfiler (4.4.4) [30]. Furthermore, a comprehensive gene set enrichment analysis (GSEA) was performed on all genes using the GSEA function in the R package clusterProfiler (4.4.4) [30]. The *p*-value was utilized as a screening index, and *p*-values not exceeding 0.05 denoted significant pathway enrichment.

**Figure 2:**
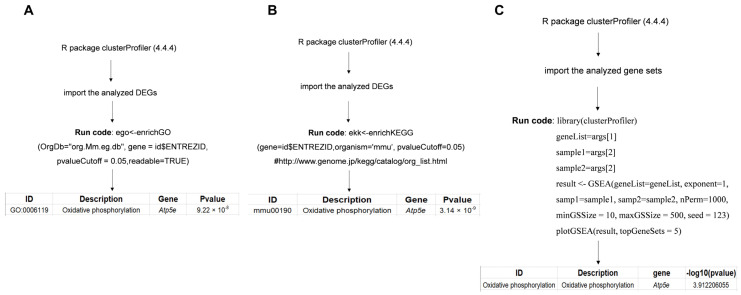
A flowchart of Gene Ontology (GO) analysis, Kyoto Encyclopedia of Genes and Genomes (KEGG) analysis, and gene set enrichment analysis (GSEA). (**A**) The steps for conducting a GO analysis; (**B**) the steps for conducting a KEGG analysis; (**C**) the steps for conducting GSEA.

The steps for conducting the GO analysis, KEGG analysis, and GSEA are shown in Figure 2. The R package was opened; then, the analyzed DEGs (GO and KEGG) or all genes (GSEA) were imported, and the code for GO analysis (Figure 2A), KEGG analysis (Figure 2B), or GSEA (Figure 2C) was run.

## 3. Statistical Analysis

Data from the behavioral tests were analyzed with the SPSS 26 software (IBM, Armonk, NY, USA), plotted with GraphPad Prism Version 9.5 (San Diego, CA, USA), and presented as the mean ± SEM. A significance level of *p* < 0.05 was applied, with differences among groups being determined via one-way ANOVA followed by Tukey’s post hoc test. The statistical analysis of the snRNA-seq data and functional enrichment was described in the previous section.

## 4. Results

### 4.1. CBD Mitigated All PTSD-like Symptoms in the SPS+S Mice Model

**Figure 3:**
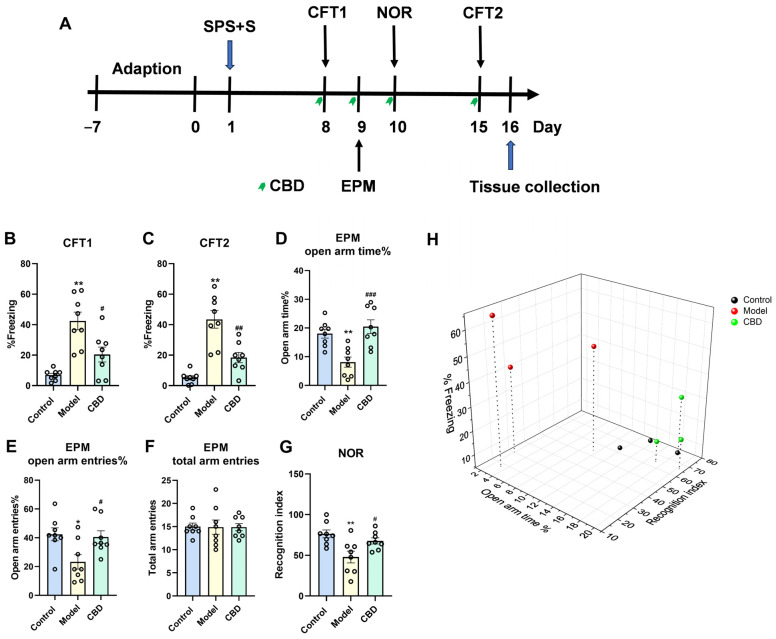
CBD showed anti-PTSD effects in the SPS+S PTSD model. (**A**) Experimental timeline and treatment schedule for the SPS+S-induced mouse model of PTSD. (**B**,**C**) On day 8 and day 15, CBD significantly reduced the contextual freezing behavior in the CFT. (**D**–**F**) On day 9, CBD reversed the decreased open arm time and open arm entries without influencing the total number of arm entries in the EPM test. (**G**) CBD reversed the decreased recognition index in the NOR test. (**H**) Three-dimensional behavioral scatterplots of three animals per group selected from the control, model, and CBD groups for single-nucleus RNA sequencing (snRNA-seq). The results are presented as the mean ± S.E.M. One-way analysis of variance (ANOVA) was conducted and was subsequently complemented by a Tukey post hoc test. * *p* < 0.05 and ** *p* < 0.01 compared with the control group; ^#^ *p* < 0.05, ^##^ *p* < 0.01, and ^###^ *p* < 0.001 compared with the model group; n = 8.

The results showed that our procedure modeled most of the PTSD-like behaviors, which were manifested by the increased freezing time of the model group on day 8 and day 15 in the CFT test, the decreased time of staying in the open arms and the times of open arm entries in the EPM test, and the decreased RI in the NOR test (Figure 3A). In the CFT, CBD significantly reversed the SPS+S-induced increase in the contextual freezing response on day 8 (F_2,21_ = 16.220, *p* < 0.001, n = 8, Figure 3B) and day 15 (F_2,21_ = 24.579, *p* < 0.001, n = 8, Figure 3C). In the EPM test, CBD significantly increased the time (F_2,21_ = 11.50, *p* < 0.001, n = 8, Figure 3D) and entries (F_2,21_ = 5.239, *p* = 0.014, n = 8, Figure 3E) into the open arms compared with those of the model group. There was no difference in the total arm entries among the three groups (Figure 3F), which indicated that neither the SPS+S procedure nor CBD administration would affect the basal locomotor activity of mice. In the NOR test, CBD significantly increased the RI compared with that of the model group (F_2,21_ = 7.040, *p* = 0.005, n = 8, Figure 3G). Based on the freezing time, open arm time, and recognition index from the above experiments, three mice from each group (control, model, and CBD) were selected for subsequent snRNA-seq. The three mice in the model group exhibited a significantly prolonged freezing time, reduced open arm time, and lower recognition index compared with those of the three mice in the control group. Conversely, the three mice in the CBD treatment group demonstrated a decreased freezing time, increased open arm time, and higher recognition index when compared with those in the model group, which indicated that our selected mice exhibited distinct behavioral performance across various dimensions, effectively representing the characteristics of the control, model, and CBD groups (Figure 3H, n = 3).

### 4.2. Analysis of Differentially Expressed Genes in the PTSD Mice

**Figure 4:**
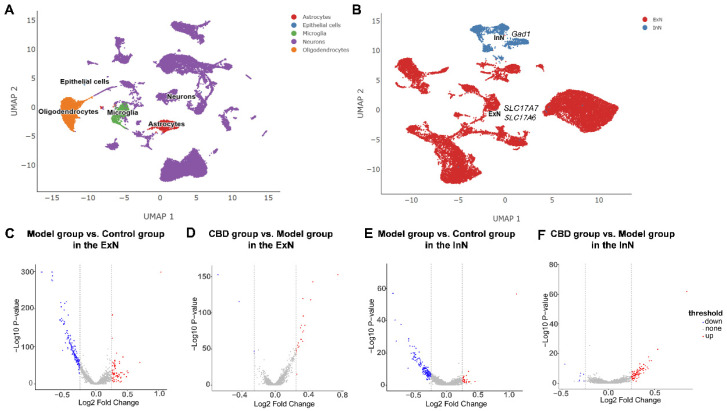
Cell type annotations and single-nucleus RNA sequencing analysis of differentially expressed genes (DEGs). (**A**) UMAP visualization of subclusters in the hippocampus. (**B**) UMAP visualization of excitatory neurons (ExNs) and inhibitory neurons (InNs) in the hippocampus. (**C**–**F**) Volcano map analysis of all DEGs in the ExNs and InNs from the control group, model group, and CBD group; heightened expression is denoted in red and diminished expression is signified by blue.

The hippocampus was selected from mice in the control, model, and CBD groups, and it was profiled using droplet-based snRNA-seq. The sequencing metrics did not differ among the groups. By utilizing graph clustering, 35 unique cell populations were discerned and characterized into cellular categories and subclasses by referencing well-acknowledged markers [31,32,33]. These 35 clusters were further classified into five major cellular categories (Figure 4A): neurons, astrocytes, oligodendrocytes, microglia, and endothelial cells. Among all of the analyzed cells, neurons constituted the largest proportion (68.4%). We focused on the neurons in this study, and these neurons were then annotated into two clusters: excitatory neurons (ExNs) and inhibitory neurons (InNs) (Figure 4B). The genes used for the neurons type annotations were solute carrier family 17 member 7 (*SLC17A7*) and solute carrier family 17 member 6 (*SLC17A6*) for ExNs, and glutamate decarboxylase 1 (*GAD1*) for InNs [34,35]. The DEGs of the ExNs and InNs in the hippocampus of mice from the control group, model group, and CBD group were then identified. In the ExNs, as shown in Figure 4C, comparing the model group to the control group, there were 68 distinct upregulated genes and 158 downregulated genes, and when the CBD group was contrasted with the model group, an observation of 21 upregulated and 4 downregulated genes emerged (Figure 4D). In the InNs, when the model group was compared with the control group, there were 20 distinct upregulated genes and 147 downregulated genes (Figure 4E), and when the CBD group was contrasted with the model group, an observation of 72 upregulated and 7 downregulated genes emerged (Figure 4F). From the results, we can see that, in the model group, for both ExNs and InNs, there were far more downregulated genes than upregulated genes. Therefore, we focused on these downregulated genes in the following analysis.

### 4.3. Functional Enrichment Analysis of ExNs in PTSD Mice

**Figure 5:**
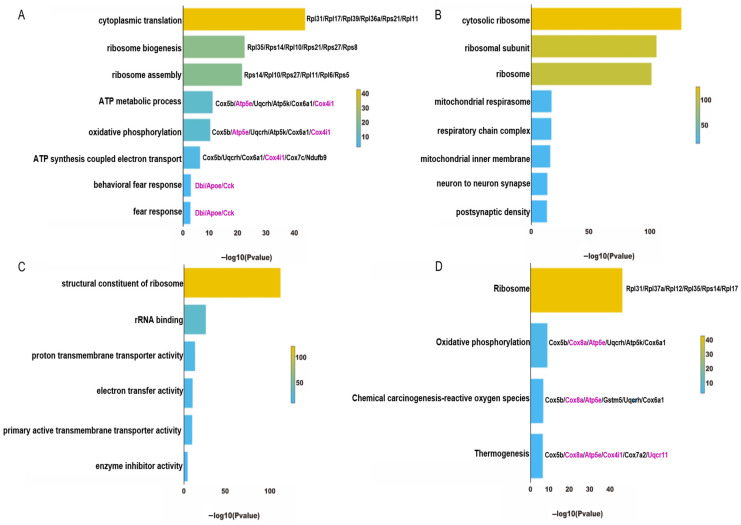
Analysis of downregulated DEGs from the GO and KEGG analyses in the ExNs of PTSD mice. (**A**) The enriched biological process pathways according to the GO analysis. (**B**) The enriched cellular component pathways according to the GO analysis. (**C**) The enriched molecular function pathways according to the GO analysis. (**D**) The KEGG pathway enrichments derived from the analysis. Genes highlighted in purple are representative genes that have a high frequency of occurrence.

GO analysis is commonly used to provide a comprehensive description of gene attributes in biological organisms, including biological processes, cellular components, and molecular function. In the ExNs, among the 158 downregulated genes in the model group, the eight pathways listed in biological processes were mainly associated with protein translation, energy metabolism, and fear response (Figure 5A). The first three pathways were related to protein translation, the central three routes pertained to the mitochondrial production of energy, and the bottom two pathways were related to fear response. In terms of cellular components, the genes related to protein translation were mainly expressed on ribosomes and their subunits; the genes related to energy metabolism were mainly located on the mitochondrial inner membrane and respiratory chain complex; the genes related to fear response were mainly expressed on synapses and axons (Figure 5B). In terms of molecular functions, the main functions of these downregulated genes included participating in ribosome components, electron transfer activity, and so on (Figure 5C). In the KEGG pathway analysis, which was consistent with the GO enrichment analysis, the downregulated genes in the model group were mainly enriched in four pathways, namely the ribosome pathway, oxidative phosphorylation pathway, reactive oxygen species pathway, and thermogenesis pathway (Figure 5D).

### 4.4. Functional Enrichment Analysis of InNs in PTSD Mice

**Figure 6:**
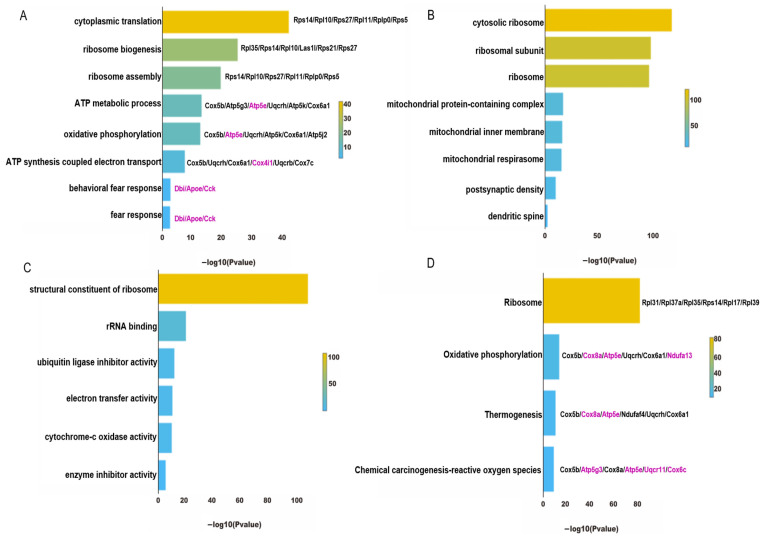
Analysis of the downregulated DEGs from the GO and KEGG analyses in the InNs of PTSD mice. (**A**) The enriched biological process pathways according to the GO analysis. (**B**) The enriched cellular component pathways according to the GO analysis. (**C**) The enriched molecular function pathways according to the GO analysis. (**D**) The KEGG pathway enrichments derived from the analysis. Genes highlighted in purple are representative genes that have a high frequency of occurrence.

In the InNs, the downregulated genes in the enriched pathways from the GO and KEGG analysis for the model group were similar to those in the ExNs. The GO analysis showed that the downregulated genes in the model group were also involved in protein translation, energy metabolism, and fear response (Figure 6A–C). In the KEGG pathway analysis, the downregulated genes in the model group were mainly enriched in four pathways, namely the ribosomal pathway, oxidative phosphorylation pathway, reactive oxygen species pathway, and thermogenesis pathway (Figure 6D).

### 4.5. Identification and Functional Enrichment Analysis of Core Genes from ExNs and InNs Involved in CBD’s Anti-PTSD Effect

**Figure 7:**
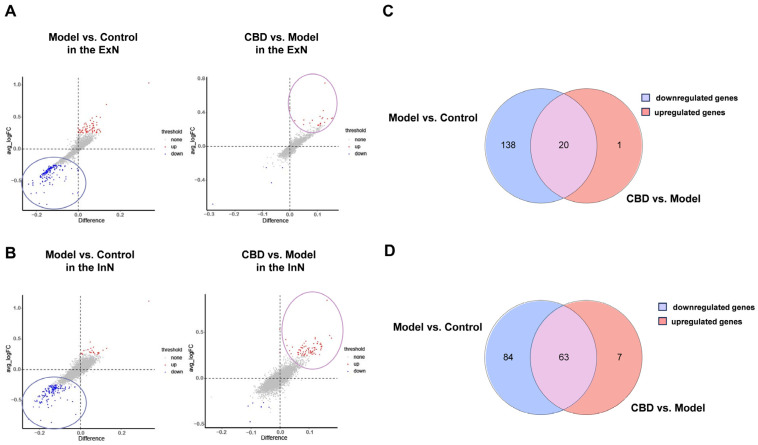
Single-nucleus RNA sequencing analysis of DEGs and overlapping DEGs in three groups in the ExNs and InNs. (**A**) DEGs of the three groups were obtained in the ExNs. (**B**) DEGs of the three groups were obtained in the InNs. (**C**) Twenty overlapping genes of downregulated genes in the model group and upregulated genes in the CBD group were obtained in the ExNs. (**D**) Sixty-three overlapping genes of downregulated genes in the model group and upregulated genes in the CBD group were obtained in the InNs. Heightened expression is denoted in red, and diminished expression is signified by blue.

To uncover the pivotal and potential targets for the protective efficacy of CBD in the treatment of PTSD, we identified genes exhibiting biphasic alterations in both the group modeling PTSD and the group undergoing CBD therapy. As shown in Figure 7A,B, the CBD group exhibited a notable restriction in the number of downregulated genes within both the ExNs and InNs, and this was accompanied by a scarcity of gene enrichment pathways. Therefore, our focus was directed toward analyzing the downregulated genes in the model group and the upregulated genes in the CBD group. In the ExNs, a total of 20 genes were discovered to exhibit biphasic changes in both the PTSD model group and the CBD treatment group. Specifically, these genes were found to be downregulated in the model group vs. the control group and subsequently upregulated in response to CBD treatment (Figure 7C). In the InNs, a total of 63 genes were found to be concurrently upregulated in the CBD group relative to the model group, and they were downregulated in the model group relative to the CBD group (Figure 7D). Because the number of overlapping genes in Figure 7C was limited to 20, and they were primarily associated with the ribosome pathway, we considered the 63 overlapping genes in Figure 7D as the pivotal genes for CBD’s action against PTSD. Subsequently, a thorough GO and KEGG pathway enrichment analysis was conducted to unravel the pharmacological principles underlying CBD’s therapeutic effects on PTSD.

**Figure 8:**
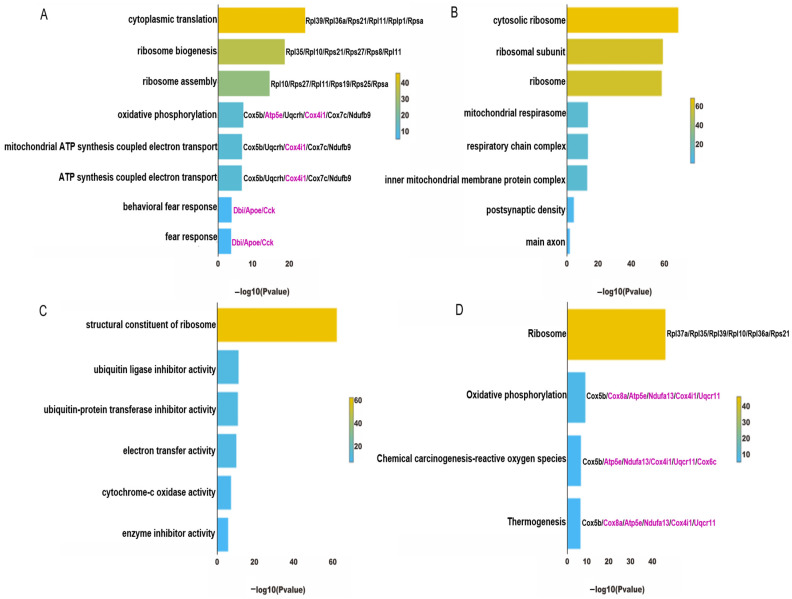
Analysis of sixty-three overlapping DEGs from the GO and KEGG analyses in the InNs of PTSD mice. (**A**) The enriched biological process pathways according to the GO analysis. (**B**) The enriched cellular component pathways according to the GO analysis. (**C**) The enriched molecular function pathways according to the GO analysis. (**D**) The KEGG pathway enrichments derived from the analysis. Genes highlighted in purple are representative genes that have a high frequency of occurrence.

The GO enrichment analysis revealed that the biological process of CBD against PTSD was linked to protein translation, energy metabolism, and fear response (Figure 8A); the cellular components of CBD’s action against PTSD were related to ribosomal subunits, the respiratory chain complex, and synapses (Figure 8B); the molecular function of CBD in the treatment of PTSD included ribosome components, electron transfer activity, and so on (Figure 8C). Furthermore, the major signaling pathways emerging from the KEGG enrichment analysis were primarily associated with four distinct pathways, namely the ribosome pathway, oxidative phosphorylation pathway, reactive oxygen species pathway, and thermogenesis pathway (Figure 8D).

### 4.6. Gene Set Enrichment Analysis (GSEA) for CBD’s Anti-PTSD Effect in the ExNs and InNs

**Figure 9:**
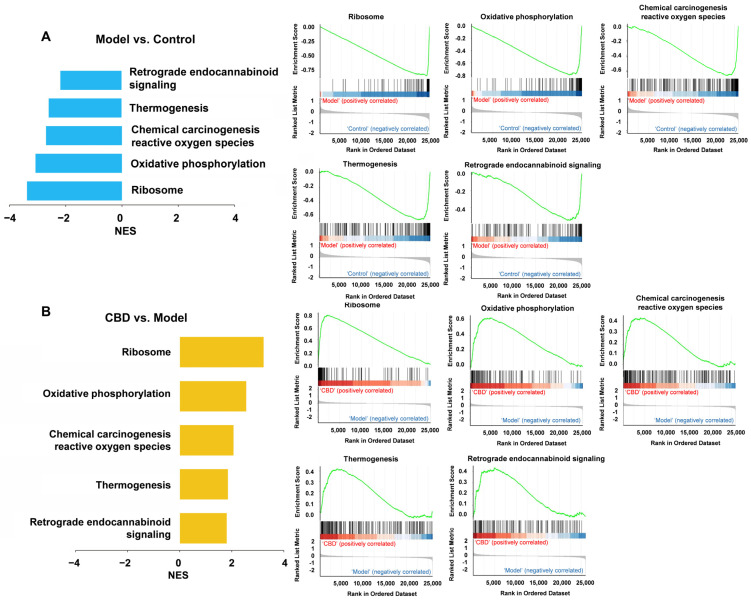
GSEA analysis of all genes in the ExNs from the control group, model group, and CBD group. (**A**) GSEA analysis of all genes of the model group vs. control group. The green line represents the running enrichment score (ES) as the analysis moves down the ranked list. The value at the peak is the final ES. Genes enriched in the model group are depicted as positive ES (red), and genes enriched in the control group are depicted as negative ES in blue. (**B**) GSEA analysis of all genes of the CBD group vs. model group. The green line represents the running ES as the analysis moves down the ranked list. The value at the peak is the final ES. Genes enriched in the CBD group are depicted as positive ES (red), and genes enriched in the model group are depicted as negative ES in blue. NES = normalized enrichment score.

**Figure 10:**
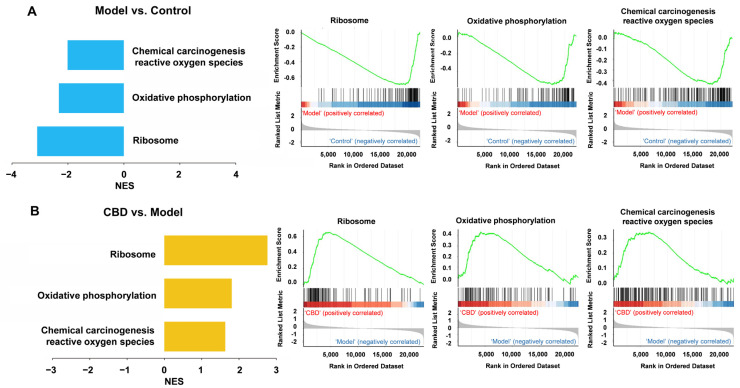
GSEA analysis of all genes in the InNs from the control group, model group, and CBD group. (**A**) GSEA analysis of all genes of the model group vs. the control group. (**B**) GSEA analysis of all genes of the CBD group vs. the model group. The green line represents the running ES as the analysis moves down the ranked list. The value at the peak is the final ES. Genes enriched in the model group are depicted as positive ES (red), genes enriched in the control group are depicted as negative ES in blue. (**B**) GSEA analysis of all genes of the CBD group vs. model group. The green line represents ES as the analysis moves down the ranked list. The value at the peak is the final ES. Genes enriched in the CBD group are depicted as positive ES (red), and genes enriched in the model group are depicted as negative ES in blue. NES = normalized enrichment score.

GSEA uses all genes instead of only differentially expressed genes to find the functional gene sets that are not significantly different but tend to be consistent in genes’ differential expression trends, and it can be used to determine whether the corresponding pathway is activated or inhibited. Therefore, GSEA was used to complement and expand the information from the GO and KEGG analyses. In the ExNs, the GSEA results showed that the model group was positively correlated with the ribosome pathway, oxidative phosphorylation pathway, reactive oxygen species pathway, thermogenesis pathway, and endocannabinoid signaling, where the genes were downregulated (Figure 9A). The CBD group was positively correlated with the ribosome pathway, oxidative phosphorylation pathway, reactive oxygen species pathway, thermogenesis pathway, and endocannabinoid signaling, where the genes were upregulated (Figure 9B). It is worth noting that retrograde endocannabinoid signaling was identified as being suppressed in the PTSD model group according to the GSEA. In the InNs, the GSEA results showed that the model group was positively correlated with the ribosome pathway, oxidative phosphorylation pathway, and reactive oxygen species pathway, where the genes were downregulated (Figure 10A). The CBD group was positively correlated with the ribosome pathway, oxidative phosphorylation pathway, and reactive oxygen species pathway, where the genes were upregulated (Figure 10B). These results indicate that the development of PTSD mainly involved alterations in the ribosome pathway, oxidative phosphorylation pathway, reactive oxygen species pathway, thermogenesis pathway, and retrograde endocannabinoid signaling pathway, which may also be the primary targets through which CBD exerts its anti-PTSD effects.

## 5. Discussion

Impaired hippocampal function is acknowledged as a prominent pathological manifestation of PTSD, and its impairment can give rise to a range of symptoms associated with PTSD [36,37]. PTSD’s key symptom is memory disturbance, which is characterized by reduced explicit memory, disjointed recollections, and trauma-induced memory loss. This issue has been widely linked to impaired hippocampal function in numerous studies [38,39,40]. By modifying the SPS PTSD model, we successfully reproduced a majority of the symptoms seen in human PTSD subjects in mice and found that CBD can effectively alleviate these PTSD-like behaviors. Here, by using this model, we report the first single-cell transcriptomic characterization of the hippocampus of mice with PTSD and identify potential molecular and pathway correlations underlying the anti-PTSD effects of CBD. Although the read-out of the genetic analysis might be limited as it is derived from single-cell analysis, the results point to genes and pathways that might be important to the understanding of the molecular mechanisms behind PTSD and CBD treatment.

Ideal animal models provide a solid foundation for the study of the pathology of mental disorders. The SPS model, a classic model of PTSD, was first established by Liberzon I et al. in 1997 using rats [41]; it involves three stress paradigms—restraint stress to simulate psychological stress, forced swimming to simulate physiological stress, and ether-simulated endocrine stress. These procedures guaranteed PTSD-like behaviors in rats for a prolonged period of time. However, it is difficult to reproduce this model in mice with the same stimulation method [42]. Several modified SPS models have appeared in other studies, such as single-cage feeding after SPS, exposing mice to dirty rat bedding after SPS, and giving electric shocks after SPS (SPS&S) [23,43,44,45]. Among these models, the SPS matching electric shocks can increase the level of stress experienced by mice. So, we added the foot shocks after SPS procedures in our model and found the long-lasting freezing behavior on day 15. While previous studies have focused only on fear behavior and anxiety-like behaviors of PTSD mice using the SPS and S model [43,44], our SPS+S model can simulate a more comprehensive heterogeneous symptom clusters of PTSD patients, making it more suitable for evaluating drug effects. In the SPS+S model, we employed CFT to assess the recurrence or avoidance behavior of individuals with PTSD, an EPM test to evaluate heightened alertness symptoms, and an NOR test to measure the cognitive impairment of PTSD patients. Following SPS+S modeling, mice in the model group exhibited a prolonged freezing response during the CFT for up to 15 days. In the EPM test, there was a significant decrease in both the number of mice entering the open arm and the percentage of time that they spent in it. Additionally, the mice in the model group displayed a reduced discrimination index in the NOR test. These findings indicate that the SPS+S mice exhibited PTSD-like behaviors, and the model demonstrated good face validity. Notably, the administration of CBD at a dose of 10 mg/kg prior to each behavioral test significantly ameliorated PTSD-like behaviors induced by SPS+S exposure, which was consistent with previous reports using pre-shock models [22,25]. The above results provide a solid foundation for our investigation of the pathological mechanisms of PTSD and the mechanism of action of CBD. However, it should be noted that CBD was reported to have anxiogenic properties in some cases. We note that, in a trace fear conditioning model, however, Uhernik et al. found that a single pre-conditioning dose of CBD (10 mg/kg) increased freezing responses [46]. CBD exhibited inconsistent effects on the regulation of fear memory at different stages, with the final outcome being greatly influenced by the timing of CBD administration and the model utilized [22,47].

### 5.1. Fear-Based Learning and Memory and the Regulation of CBD in PTSD

In this study, our primary focus was on a substantial proportion of neurons known as ExNs and InNs. It is imperative to emphasize the critical role played by the delicate balance between these two neuronal types in ensuring the normal functioning of the brain. The results of the GO and KEGG analyses showed a high degree of similarity in the downregulated DEGs between ExNs and InNs. These genes were found to be associated with the fear response, ribosome pathway, oxidative phosphorylation pathway, reactive oxygen species pathway, and thermogenesis pathway.

Fear-related impairment of learning and memory constitutes a fundamental pathological mechanism underlying PTSD. In PTSD patients, the resistance to the extinction of traumatic memories stands as a prominent factor contributing to the exacerbation of their symptoms and posing challenges in treatment. The extinction of fear memory is a learning process that entails multiple molecular mechanisms, such as protein post-translation modifications, genetic expression, and likely the synthesis of new proteins from scratch [40]. According to reports, the promotion of memory and cognition is facilitated by protein synthesis in hippocampal neurons [48]. The behavioral findings in our study indicated that PTSD-modeled mice exhibited enduring fear memory and cognitive impairment, which also successfully simulated the symptoms of PTSD patients. According to the results of the GO and KEGG analyses, genes related to the ribosome pathway in both ExNs and InNs from PTSD mice were downregulated, indicating that the protein synthesis function in the hippocampus of the PTSD mice was impaired. This finding helps to explain the deterioration in cognitive function and impaired capacity to learn about safe environments observed in individuals with PTSD. Surprisingly, genes in the InNs related to the ribosome pathway were upregulated when treated with CBD, indicating that CBD could potentially alleviate the disruption in protein synthesis in InNs, thereby manifesting its anti-PTSD properties.

In our study, biological processes related to fear response were identified by the GO analysis as being involved in the development of PTSD both in ExNs and InNs, which again conveyed the idea that this process is essential for PTSD. CBD reversed the alteration of this process in InNs. We identified three main genes in the process of fear response that were downregulated, namely diazepam binding inhibitor (*Dbi*), apolipoprotein E (*Apoe*), and cholecystokinin (*Cck*); this effect was reversed by CBD. This implies that CBD may possess a more precise impact on fear memory and may predominantly exert its influence by modulating InNs. The decrease in Dbi expression within this brain region aligned with numerous findings that suggested a connection between human panic disorder and an attenuated reaction to diazepam [49]. Overexpression of *Dbi* led to a reduction in freezing responses during the context test, and blocking *Dbi* augmented contextual fear induced by prolonged corticotropin-releasing factor activation [50,51]. *Cck*, a neuropeptide primarily localized in GABAergic neurons, is known to increase the release of γ-aminobutyric acid (GABA) in the cerebral cortex and hippocampus. Recently, Li’s laboratory discovered that time-dependent sensitized PTSD rats significantly reduced GABA levels in the prefrontal cortex and hippocampus, which were reversed by sertraline [52]. *ApoE* is an essential component of lipoprotein particles in both the brain and periphery. It was reported that polyunsaturated fatty acids’ neuronal metabolic conversion into endocannabinoids is distinctively facilitated by *ApoE3*, thus revealing the positive correlation of this gene with endocannabinoids [53]. Therefore, we deduced that the downregulation of *ApoE* led to a lower level of endocannabinoids in the model group, and the upregulation of *ApoE* by CBD treatment restored the level of endocannabinoids.

### 5.2. The Endocannabinoid System and the Regulation of CBD in PTSD

We observed a significant concurrence between the outcomes of the GSEA and the majority of the KEGG pathways, with the exception being retrograde endocannabinoid signaling, which was downregulated in the ExNs of the model group and upregulated in the CBD group. This suggested a strong correlation between alterations in the endocannabinoid system in ExNs and the onset and progression of PTSD. The endocannabinoid system comprises endogenous cannabinoids—specifically, anandamide (AEA) and 2-arachidonoylglycerol (2-AG)—along with cannabinoid receptors of types 1 and 2 (CB1 and CB2). It also encompasses enzymes that facilitate both the creation and breakdown of these endocannabinoids. Research has indicated that this system plays a pivotal role in regulating a broad spectrum of physiological processes, including pain sensation, mood regulation, immune response, and memory [54,55,56,57]. Consistent with the implications of our study, Fidelman [58] et al. suggested that AEA was decreased in CA1 of the hippocampus after shock, and that improving endocannabinoid signaling through the application of a fatty acid amide hydrolase (FAAH) inhibitor, URB597, has the potential to alleviate PTSD-like behaviors in rodents. Enhanced SPS modeled rats exhibited decreased endocannabinoid signaling, such as the downregulation of diacylglycerol lipase α and CB1R in the hippocampus. Electroacupuncture enhanced hippocampal endocannabinoid signaling to prevent PTSD-like behaviors [59]. Neural plasticity and excitability are also influenced by the endocannabinoid system. Research conducted by Reich et al. revealed that administering the CB1 receptor agonist WIN 55,212-2 at a concentration of 1 μM to animals under stress led to a notable escalation of approximately 135% in excitatory neurotransmission [60]. CBD, as an inhibitor of FAAH, can play the role of enhancing the endocannabinoid system, which was shown in the GSEA results. Therefore, targeting the endocannabinoid system as a therapeutic strategy for PTSD holds significant potential, and CBD emerges as a competitive compound that is worthy of consideration.

### 5.3. Oxidative Phosphorylation and the Regulation of CBD in PTSD

In our study, we observed a significant downregulation of oxidative phosphorylation pathway-related genes, including NADH: ubiquinone oxidoreductase subunit A13 (*Ndufa13*), ubiquinol-cytochrome c reductase, complex III subunit XI (*Uqcr11*), cytochrome c oxidase subunit 6C (*Cox6c*), cytochrome c oxidase subunit 8A (*Cox8a*), and ATP synthase epsilon chain (*Atp5e*), in both InNs and ExNs in the hippocampus of PTSD mice. Additionally, the GO analysis revealed a downregulation of energy metabolism pathways, specifically oxidative phosphorylation processes and the ATP generation linked to the electron transport chain in PTSD mice, indicating mitochondrial dysfunction. In a clinical study, PTSD patients exhibited downregulation of certain genes related to the oxidative phosphorylation pathway in the dorsolateral prefrontal cortex, including *Atp5e*, *Cox8a*, NADH: ubiquinone oxidoreductase subunit B5 (*Ndufb5*), and NADH: ubiquinone oxidoreductase core subunit S2 (*Ndufs2*) [61]. Our results align with prior research that reported mitochondrial dysfunction in the prefrontal cortex of rats with PTSD through RNA sequencing [62,63]. More than 90% of the cellular energy generation takes place within the mitochondria through a process known as oxidative phosphorylation, and it has also been found that oxidative phosphorylation is a biochemical process that occurs in the inner mitochondrial membrane; it fuels neuronal physiological functions by producing ATP energy stores [64]. In addition, cellular energy-dependent protein synthesis, such as that occurring within “ribosomes” and in “cytoplasmic translation”, is a process that can be hindered by inefficient energy metabolism [65,66]. Therefore, therapeutic interventions aimed at modulating energy metabolism pathways may yield optimal results. Henningsen et al. [67] suggested that enhanced oxidative phosphorylation, as indicated by heightened levels of cytochrome c oxidase subunit 5B (*Cox5b*), ubiquinol-cytochrome c reductase binding protein (*Uqcrb*), NADH: ubiquinone oxidoreductase core subunit S8 (*Ndufs8*), NADH: ubiquinone oxidoreductase subunit B7 (*Ndufb7*), and cytochrome c oxidase subunit 5A (*Cox5a*), constitutes a response strategy against stress. In our study, CBD upregulated the expression of oxidative-phosphorylation-related genes (*Cox5a*, *Atp5e*, *Cox5b*, etc.) in the hippocampus of PTSD mice, which may have contributed to a protective effect against the development or manifestation of symptoms associated with PTSD. In animal models of several other diseases, CBD has been demonstrated to exert therapeutic effects by increasing oxidative phosphorylation levels. Sun et al. [68] found that CBD hindered a decline in the oxygen consumption rate associated with ATP production and restored mitochondrial functionality in a model of cerebral ischemia. Ibork et al. [69] found that CBD counteracted LPS-induced metabolic disturbances and inflammation by stimulating oxidative phosphorylation in astrocytes, an effect linked to its interaction with CB1 receptors. Our findings indicate, for the first time, that CBD may alleviate PTSD by enhancing oxidative phosphorylation.

### 5.4. Oxidative Stress and the Regulation of CBD in PTSD

The reactive oxygen species (ROS) pathway exhibited significant downregulation in the model group and upregulation in the CBD group in our study. Oxidative stress refers to a cellular condition in which there is an imbalance between the production of pro-oxidant molecules, such as ROS, and the availability of cellular antioxidants, such as superoxide dismutase (SOD). In a clinical study conducted by Zieker et al. [70], the authors noted a significant decrease in the transcription levels of SOD, an essential anti-oxidative enzyme, among PTSD patients who had witnessed a catastrophic air show disaster. Delaš laboratory [71] found reduced levels of SOD and glutathione peroxidase enzymes in Croatian veterans with PTSD who were actively involved in the Homeland War. In a preclinical trial, Xie et al. [72] demonstrated that SPS-modeled mice exhibited decreased SOD activity in the hippocampus. Ebenezer et al. [73] found that rats with PTSD exhibited heightened overall ROS generation in the predator stress-induced PTSD model, specifically within the prefrontal cortex and hippocampus. Furthermore, oxidative stress has been shown to result in the downregulation of various genes, including subunit A of succinate dehydrogenase (*Sdha*), the panthenol cytochrome c oxidoreductase gene (*Uqcr*), cytochrome oxidase genes such as cytochrome c oxidase subunit 6A2 (*Cox6a2*), cytochrome c oxidase copper chaperone COX17 (*Cox17*), and ATP synthetase genes such as ATP synthase F1 subunit α (Atp5a1), ATP synthase, H+ transporting, mitochondrial Fo complex subunit C1(Atp5g1), and ATPase inhibitory factor 1 (Atpif1) [74,75], which implies a negative correlation between the expression of these genes and oxidative stress levels. The GO analysis in our study revealed decreased expression of the *Uqcr11*, *Cox6c*, and *Atp5e* genes in the model group; their expression was upregulated in response to CBD treatment. Our findings, in conjunction with those of previous reports, suggest that oxidative stress alterations play a pivotal role in the pathogenesis of PTSD. Moreover, CBD has the potential to ameliorate this process via the regulation of these genes and exert its anti-PTSD effects.

## 6. Conclusions

In summary, in the present study, a GO analysis and GSEA revealed that possible involvements in the causation of PTSD could stem from protein synthesis, oxidative phosphorylation, oxidative stress response, and the endocannabinoid system. Functional and pathway analyses of DEGs and gene sets suggested that CBD may primarily exert its anti-PTSD effects by modulating InNs via the regulation of protein synthesis, oxidative phosphorylation, oxidative stress response, and fear response and by regulating the endocannabinoid signaling of ExNs. Our study shows that the snRNA-seq dataset, despite its restricted sample size, outperformed RNA-seq datasets in detecting DEGs. This research contributes to the identification of potential therapeutic target genes in the context of PTSD progression. Furthermore, it opens new avenues for comprehension of the mechanisms underlying the anti-PTSD effects of CBD, offering innovative strategies for future investigations.

## Data Availability

Data will be made available on request due to privacy.
